# Discovery of reversing enzymes for RNA ADP-ribosylation reveals a possible defence module against toxic attack

**DOI:** 10.1093/nar/gkaf069

**Published:** 2025-02-18

**Authors:** Yang Lu, Marion Schuller, Nathan P Bullen, Petra Mikolcevic, Iva Zonjic, Roberto Raggiaschi, Andreja Mikoc, John C Whitney, Ivan Ahel

**Affiliations:** Sir William Dunn School of Pathology, University of Oxford, Oxford, OX1 3RE, United Kingdom; Sir William Dunn School of Pathology, University of Oxford, Oxford, OX1 3RE, United Kingdom; Department of Biochemistry and Biomedical Sciences, McMaster University, Hamilton, ON L8S 4K1, Canada; Division of Molecular Biology, Ruđer Bošković Institute, Zagreb, 10000, Croatia; Division of Molecular Biology, Ruđer Bošković Institute, Zagreb, 10000, Croatia; Sir William Dunn School of Pathology, University of Oxford, Oxford, OX1 3RE, United Kingdom; Division of Molecular Biology, Ruđer Bošković Institute, Zagreb, 10000, Croatia; Department of Biochemistry and Biomedical Sciences, McMaster University, Hamilton, ON L8S 4K1, Canada; Sir William Dunn School of Pathology, University of Oxford, Oxford, OX1 3RE, United Kingdom

## Abstract

Nucleic acid ADP-ribosylation and its associated enzymes involved in catalysis and hydrolysis are widespread among all kingdoms of life. Yet, its roles in mammalian and bacterial physiology including inter-/intraspecies conflicts are currently underexplored. Recently, several examples of enzymatic systems for RNA ADP-ribosylation have been identified, showing that all major types of RNA species, including messenger RNA, ribosomal RNA, and transfer RNA, can be targeted by ADP-ribosyltransferases (ARTs) which attach ADP-ribose modifications either to nucleobases, the backbone ribose, or phosphate ends. Yet little is known about the reversibility of RNA ADP-ribosylation by ADP-ribosylhydrolases belonging to the macrodomain, ARH, or NADAR superfamilies. Here, we characterize the hydrolytic activity of ADP-ribosylhydrolases on RNA species ADP-ribosylated by mammalian and bacterial ARTs. We demonstrate that NADAR ADP-ribosylhydrolases are the only hydrolase family able to reverse guanosine RNA base ADP-ribosylation while they are inactive on phosphate-end RNA ADP-ribosylation. Furthermore, we reveal that macrodomain-containing PARG enzymes are the only hydrolase type with the ability for specific and efficient reversal of 2′-hydroxyl group RNA ADP-ribosylation catalysed by *Pseudomonas aeruginosa* effector toxin RhsP2. Moreover, using the RhsP2/bacterial PARG system as an example, we demonstrate that PARG enzymes can act as protective immunity enzymes against antibacterial RNA-targeting ART toxins.

## Graphical Abstract

**Graphical Abstract:**
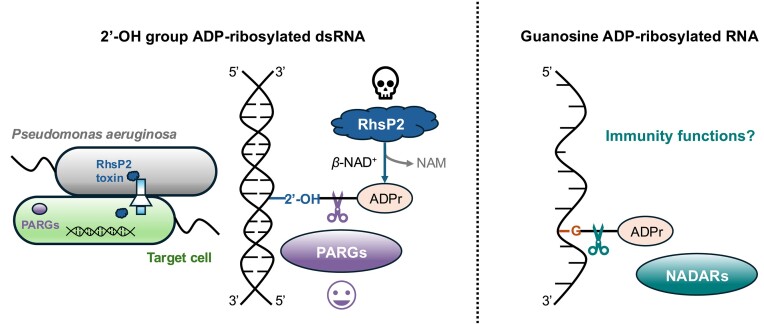

## Introduction

ADP-ribosylation is a modification of macromolecules that regulates many processes including DNA repair, transcription, bacterial metabolism, and interspecies conflicts [1–6]. ADP-ribosylation is synthesized by various ADP-ribosyltransferases (ARTs), which include bacterial toxins such as cholera toxin [7] or poly(ADP-ribose) polymerases (PARPs), the best understood group of ARTs [8]. ARTs utilize nicotinamide adenine dinucleotide (NAD^+^) as a source for the modification and covalently attach ADP-ribose to the target molecule, releasing nicotinamide in the process [9, 10]. For most ARTs, ADP-ribosylation is a readily reversible modification [11], except in cases of virulence factors such as anti-eukaryotic diphtheria toxin for which reversibility has not been confirmed yet [12]. ADP-ribosylation-reversing enzymes belong to three known protein families: the macrodomain [13, 14], ADP-ribosylhydrolases (ARH/DraG) [15, 16], and the NADAR family [17]. Both proteins and nucleic acids serve as acceptors for ADP-ribosylation [1, 18]. While protein ADP-ribosylation is better understood, particularly in mammalian systems [19–23], ADP-ribosylation of nucleic acids has been much less studied [18, 24].

The ADP-ribosylation of DNA (DNA-ADPr) has been established as physiologically relevant in bacterial systems [4]. For example, DarTG toxin–antitoxin systems that mediate tightly controlled reversible DNA-ADPr targeting thymidine or guanosine bases [17, 25, 26] are used to control bacterial replication and growth [27] or to defend against phages [28]. DarT toxins are ARTs that are highly diverged from the mammalian PARPs [27], while the DNA-ADPr-reversing antitoxins belong either to the macrodomain [14, 25] or to the NADAR family of hydrolases [17, 29]. Other toxins from bacteria and eukaryotes can target guanosine bases in DNA for ADP-ribosylation such as Scabin from *Streptomyces scabies* and pierisin-1 from cabbage butterfly [30–32]. Moreover, recent data suggest that endogenous reversible DNA-ADPr may also exist in mammals [33–37] where it is, for instance, suggested to play a role in telomere replication ensuring genome stability [35].

RNA modification with ADP-ribose is also emerging with the 3′- and 5′-phosphorylated ends [38], guanosine bases [39], and the ribose 2′-hydroxyl group (2′-OH) [40] being identified as target sites. The physiological relevance of phosphate group ADP-ribosylation is unclear. It is hypothesized to have a specific regulatory function such as non-canonical capping of RNA ends, though could also be a product of erroneous, promiscuous activity of ARTs from bacteria and higher organisms including Tpt1/KptA, PARP10, and PARP14 [10, 38, 41, 42]. Similarly to DNA ADP-ribosylated at phosphate ends, ADP-ribosylation of phosphorylated RNA ends is known to be reversible by many different hydrolases including PARG, TARG1, ARH3, MacroD1 and MacroD2, and macrodomain 1 of PARP9 and PARP14 [38, 42, 43].

ADP-ribose modifications of RNA were also shown to occur at guanosine bases which are synthesized by some ARTs from the pierisin family that are thought to irreversibly modify their targets. SCO5461, also known as ScARP (*Streptomyces coelicolor* ADP-ribosylating protein) [39], is a member of this protein family that can *in vitro* ADP-ribosylate guanosine bases in RNA/DNA as well as guanosine and guanine mononucleotides [39]. ScARP is a secreted toxin that is probably used in interspecies conflicts and has been shown to be involved in the regulation of morphological differentiation and antibiotic production [44]. Furthermore, RhsP2 is a recently discovered toxin which is secreted by *Pseudomonas aeruginosa* via type VI secretion system and belongs to the rearrangement hot spot family of polymorphic toxins. Its C-terminal toxin domain is an ART that modifies ribose 2′-OH of various double-stranded RNA (dsRNA) species leading to inhibition of translation and cell death in competitor bacteria [40].

To date, characterized examples of ADP-ribosylation of RNA nucleobases and hydroxyl groups are consequences of toxic attacks employed in intra- and interspecies conflicts. However, it remains unknown whether ADP-ribosylhydrolases, which act on modified DNA and protein, also exist to counteract modifications generated by RNA-targeting ART toxins. In this study, we identified enzymes from the macrodomain and NADAR families that can specifically reverse RNA ADP-ribosylation modifications. Notably, macrodomain-containing human and bacterial PARG enzymes were found to hydrolyse RhsP2-catalysed 2′-OH RNA ADP-ribosylation, while NADAR enzymes efficiently remove ADP-ribosylation marks from guanosine bases in RNA catalysed by ScARP. Furthermore, using the RhsP2/PARG system as an example, we find that PARG-type macrodomain family members can protect bacteria against toxicity mediated by RNA-targeting ART toxins, suggesting those enzymes may provide immunity during bacterial competition.

## Materials and methods

### Materials

DNA and RNA substrates were synthesized by Thermo Scientific or Integrated DNA Technologies (IDT). Cy3-labelled oligonucleotides were purchased from IDT. Oligonucleotides and primers used in this study are listed in Supplementary Tables S1 and S2.

### Bacterial strains and culture conditions

*Escherichia coli* strain XL1-Blue was used for the RhsP2/PARG toxicity experiments. Cultures were grown at 37°C in LB medium supplemented with 200 μg/ml trimethoprim and 15 μg/ml gentamicin for the maintenance of pSCrhaB2-CV and pPSV39-CV, respectively. Solid media contained 1% (w/v) agar. Where appropriate, cultures we additionally supplemented with 0.2% (w/v) l-rhamnose for the induction of genes cloned into pScrhaB2-CV and 250 μM isopropyl β-d-1-thiogalactopyranoside (IPTG) for the induction of genes cloned into pPSV39-CV. Bacterial strains used in this study are listed in Supplementary Table S3.

### DNA manipulation and plasmid construction

All primers were synthesized and purified by IDT. Phusion polymerase, restriction enzymes, and T4 ligase were obtained from New England Biolabs. Plasmids were constructed through standard restriction enzyme-based cloning procedures.

### Bacterial toxicity assays

#### RhsP2_tox_ and PARG transformation

Due to the extreme toxicity of the RhsP2 C-terminal toxin domain (residues 1445-CT), we were unable to generate an RhsP2_tox_ expression plasmid. To circumvent this, we cloned an active site variant (Y1524A) that was previously shown to have attenuated activity [40] into pScrhaB2-CV. We then transformed 500 ng of this plasmid into chemically competent *E. coli* XL1-Blue strains harbouring either empty pPSV39, rhsI2, SCO0909, or DRB0099 that were previously grown in the presence of IPTG to induce the expression of the PARGs or RhsI2. Transformations were then plated on LB agar supplemented with 0.5 mM IPTG, 200 μg/ml trimethoprim, and 15 μg/ml gentamicin.

#### Growth curves

*Escherichia coli* cells harbouring either empty vectors or RhsP2_Y1524A_ C-terminus and the indicated PARGs were grown overnight (O/N) and back-diluted into LB broth supplemented with 200 μg/ml trimethoprim and 15 μg/ml gentamicin. Cultures were grown until they reached an OD_600_ of 0.3 upon which protein expression was induced with the addition of 0.2% (w/v) l-rhamnose and 250 μM IPTG. *P*-values were determined by one-way ANOVA using GraphPad Prism version 10.3.

### Gel-shift ADP-ribosylation activity assays

Oligo ADP-ribosylation reactions were conducted in a buffer solution comprising 20 mM HEPES–KOH (pH 7.6), 50 mM KCl, 5 mM MgCl_2_, and 1 mM DTT. For ADP-ribosylation, the reactions were performed in a total volume of 10 μl, incubating ARTs [1 μM of all transferases except 3 μM TRPT1 (human KptA)] with oligonucleotides (50mer RNA oligo: 3 μM; PolyU-G/PolyU-A: 40 μM; 5′ and 3′-Phos-DNA/RNA: 1 μM; and 5′-Phos-Ex21-Cy3: 0.3 μM) and an excess of β-NAD^+^ (1 mM) at 37°C for 60 min. SCO3953 modification reactions were performed with 1.5 μM transferase at 30°C. The reactions were terminated by heating the samples at 95°C for 5 min. Following this, for de-modification assays catalysed by hydrolases, the ADP-ribosylated samples were incubated with either buffer as a control (+) or 1 μM (or 2 μM were specified in titration experiments) of the specified hydrolase at 37°C for 30 min. All transferases and hydrolases used in this assay were analysed by sodium dodecyl sulfate–polyacrylamide gel electrophoresis (SDS–PAGE), as shown in Supplementary Fig. S6. The final products were analysed by separating them on denaturing urea polyacrylamide gels in 1× Tris/Borate/EDTA (TBE) buffer. Before loading, 10 μl urea loading dye containing 10 mM Tris–HCl (pH 8.0), 10 mM ethylenediaminetetraacetic acid, and 4 M urea was added into the samples, followed by an incubation at 95°C for 5 min. Then, 10 μl of each sample was loaded onto the urea gel for electrophoresis, and the oligos were visualized under 340-nm ultraviolet light after staining with SYBR Gold Nucleic Acid Gel Stain (Invitrogen), for unlabelled oligos, or under 532-nm laser excitation using the Molecular Imager PharosFX system (Bio-Rad) for Cy3-labelled oligos. Unless stated otherwise in the figure legend, gel-shift ADP-ribosylation activity assays were performed with unlabelled oligonucleotides.

If not specifically stated, negative controls (−) are samples treated identically to all other samples, except that buffer is added instead of transferase enzyme in the reactions during the ADP-ribosylation step, allowing for the comparison of unmodified oligos as an internal reference to the ADP-ribosylated oligos. Samples indicated as positive controls (+) are also treated the same as all other samples, except they are supplemented only with buffer and without the addition of hydrolase enzyme for the de-modification step. This allows for the comparison of ADP-ribosylated oligos as an internal reference to the unmodified oligos.

For all panels, representative results of two independent experiments are shown (please also refer to Supplementary Fig. S7).

### Recombinant protein expression and purification

*Escherichia coli* Rosetta strain BL21(DE3) was transformed with bacterial PARG enzymes and grown at 37°C in LB (Sigma) supplemented with 50 μg/ml of kanamycin and 35 μg/ml of chloramphenicol. After reaching an OD_600_ of 0.5, the temperature was lowered to 18°C prior to induction of protein expression O/N by adding 0.5 mM IPTG. Harvested cells were resuspended in lysis buffer [50 mM HEPES (pH 7.4), 500 mM NaCl, 5% glycerol, 20 mM imidazole, 0.5 mM tris(2-carboxyethyl)phosphine (TCEP), cOmplete EDTA-free protease inhibitors (Roche)] and stored at −20°C until purification.

For protein purification, pellets were gently thawed and lysed by high-pressure homogenization. DNA was digested using Benzonase Nuclease (Merck Life Science). Proteins were purified by immobilized metal affinity chromatography using Ni-Sepharose resin (GE Healthcare) and eluted stepwise in binding buffer containing 40–500 mM imidazole. Proteins were stored at −80°C after O/N dialysis into buffer consisting of 25 mM HEPES (pH 7.5), 300 mM NaCl, 5% glycerol, and 0.5 mM TCEP.

### Western blotting

*Escherichia coli* cells harbouring either empty vectors or RhsP2_Y1524A_ C-terminus and the indicated PARG were grown O/N and back-diluted 1/50 into 5 ml of LB broth supplemented with 200 μg/ml trimethoprim and 15 μg/ml gentamicin. Cultures were grown until they reached an OD_600nm_ of 0.6, upon which 0.2% (w/v) l-rhamnose and 500 μM IPTG were added to the cultures. After 30 min, the cells were pelleted by centrifugation and resuspended in 100 μl of 1× phosphate-buffered saline. The resuspended cells were then mixed 1:1 with Laemmli buffer and then boiled for 10 min. Samples were then centrifuged at 16 000 × *g* and 5 μl of the resulting supernatant was loaded onto a 10% SDS–PAGE gel. The gel was run for 55 min at 155 V, and total protein was wet transferred to a nitrocellulose membrane using a mini Trans-Blot electrophoretic system (Bio-Rad). Following the transfer, the blot was blocked in phosphate-buffered saline with 1% (v/v) Tween^®^ 20 (PBS-T) containing 5% (w/v) non-fat milk for 1 h and then washed three times with 10 ml of PBS-T. A total of 10 ml of PBS-T containing a 1/5000 dilution of anti-VSV-G (MilliporeSigma, V4888) was then added to the blot followed by an O/N incubation at 4°C. The blot was then washed three times with PBS-T and incubated with a 1/5000 dilution anti-rabbit HRP conjugate. After 1 h, the blot was washed three times with PBS-T and developed by the addition of Clarity Max ECL substrate. The resulting chemiluminescence was visualized using a ChemiDoc Touch Imaging System (Bio-Rad).

### Phylogenetic tree for PARG and macrodomain ADP-ribosylhydrolases

PARG_cat (PARG), DUF2263 (bactPARG), and macrodomains of the selected proteins were aligned using the MUSCLE algorithm [45]. The evolutionary history was inferred using the maximum likelihood method and Whelan and Goldman model [46]. A discrete gamma distribution was used to model evolutionary rate differences among sites. The tree is drawn to scale, with branch lengths measured in the number of substitutions per site. The evolutionary analyses were performed in MEGA X [47].

## Results

### NADARs reverse guanosine RNA ADP-ribosylation

**Figure 1.**
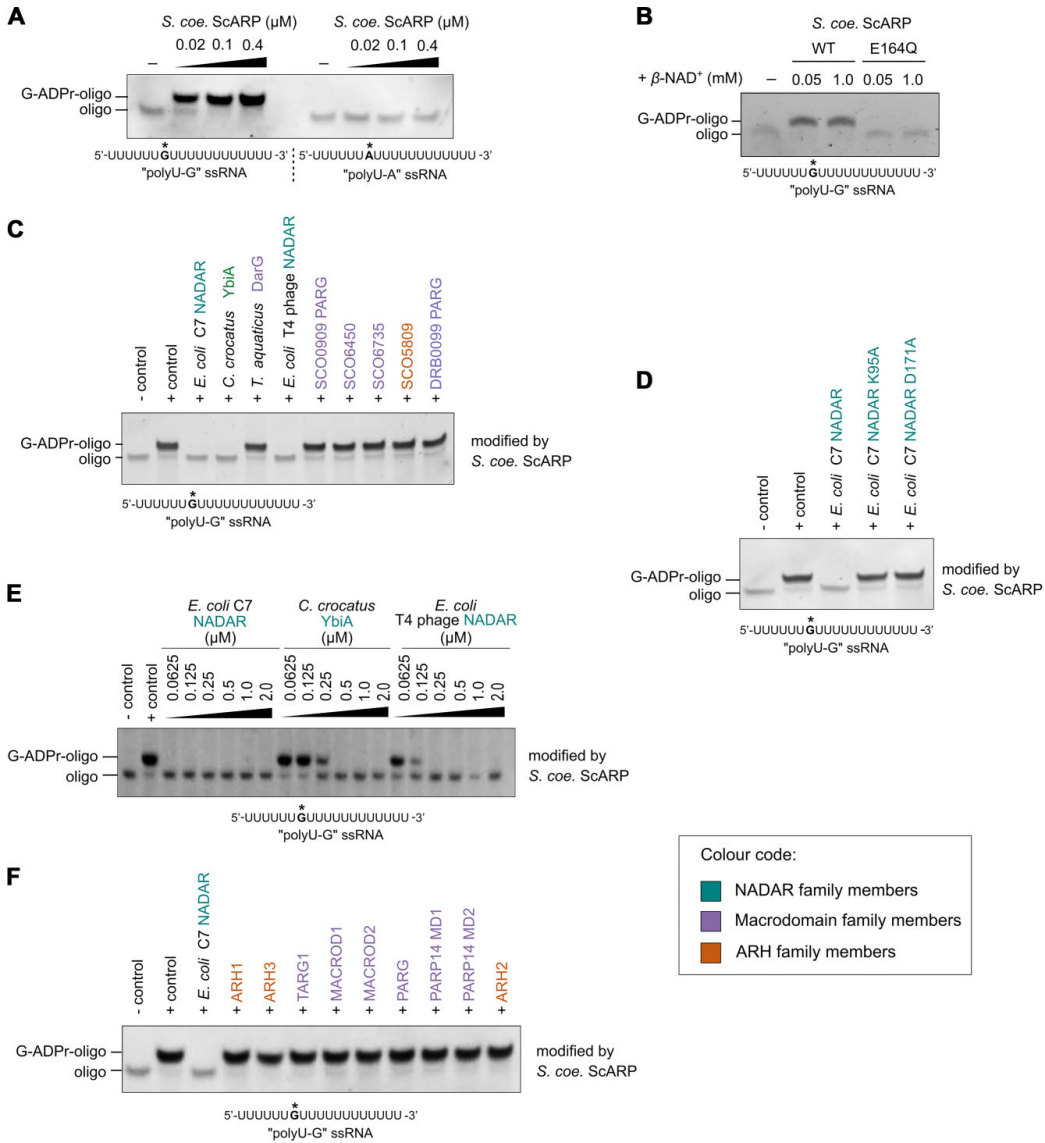
Reversal of guanosine RNA ADP-ribosylation by NADARs. (**A**) *S. coelicolor* ScARP ADP-ribosylates guanosines in RNA oligonucleotides. *In vitro* ADP-ribosylation assays were performed using the substrates shown below the gel image with the tested guanosine nucleotides targeted for ADP-ribosylation by ScARP highlighted with an asterisk. (**B**) *S. coelicolor* ScARP wild-type ADP-ribosylates ‘PolyU-G’ compared with ScARP catalytic mutant. (**C**) NADARs reverse guanosine RNA ADP-ribosylation catalysed by ScARP compared with bacterial macrodomain and ARH-like enzymes. (**D**) *E. coli* C7 NADAR wild-type hydrolyses guanosine RNA ADP-ribosylation compared with catalytically inactive mutants. (**E**) Comparison of hydrolytic efficiencies of NADAR enzymes active on guanosine RNA ADP-ribosylation by enzyme titration. (**F**) Human ADP-ribosylhydrolases are not able to reverse guanosine RNA ADP-ribosylation. The colour code for NADAR, macrodomain, and ARH family members is shown in the inset bottom right.

We first sought to uncover whether any of the known ADP-ribosylhydrolases have the ability to reverse ADP-ribosylation of guanosine bases in RNA. For this, we first established a well-defined synthetic ADP-ribosylated oligonucleotide substrate. Since *S. coelicolor* SCO5461, i.e. ScARP, protein was previously shown to ADP-ribosylate yeast transfer RNA (tRNA) [39], we tested its ability to modify a poly-U-oligonucleotide containing a single guanosine (‘PolyU-G’) using our established gel-shift-based assay [17]. We observed efficient modification of this substrate by ScARP by revealing a shift corresponding to a single ADP-ribose modification attached when compared with the unmodified control oligonucleotide (Fig. 1A). Of note, the presence of the ADP-ribose modification on the pyrimidine-oligonucleotide ‘PolyU-G’ enhances the SYBR dye staining of the oligo and its visualization (Supplementary Fig. S1). Notably, this oligonucleotide could not be modified by the ScARP catalytically inactive mutant ScARP E164Q (Fig. 1B). Furthermore, when the guanosine nucleotide was replaced by an adenosine, the RNA oligonucleotide (‘PolyU-A’) could not be modified by ScARP confirming specificity of the reaction for guanosine RNA ADP-ribosylation (Fig. 1A). Having established the substrate for the hydrolases, we next tested a panel of bacterial ADP-ribosylhydrolases—representing the three superfamilies known for ADP-ribosylation reversal—against guanosine ADP-ribosylated RNA. This included several NADAR family proteins, that were previously shown to hydrolyse ADP-ribosylation modifications from guanines in DNA [17, 29], and bacterial representatives from previously characterized ADP-ribosylhydrolases of the macrodomain family from *Streptomyces* species [38, 48]. Furthermore, as an ARH-like representative, we utilized the uncharacterized protein from *S. coelicolor* SCO5809 that is homologous to human ARH3 protein [49]. We observed that all NADAR family representatives reversed guanosine ADP-ribosylated RNA, whereas the macrodomain- and ARH-like hydrolases showed no activity (Fig. 1C). Catalytic mutations in *E. coli* C7 NADAR [17] abolished ADP-ribosylation removal from the guanosine bases as expected (Fig. 1D). Furthermore, we observed that *E. coli* C7 NADAR showed significantly higher hydrolytic activity compared with T4 phage NADAR and *E. coli* YbiA (Fig. 1E) which may be related to the nucleic acid-binding capabilities *E. coli* C7 NADAR increasing the affinity for this substrate. Apparent NADAR orthologues are not present in humans and other vertebrate species [17, 29]; however, we analysed a panel of characterized human enzymes representing macrodomain and ARH superfamily members. Notably, none of these human proteins were able to reverse guanosine ADP-ribosylated RNA (Fig. 1F). Altogether, we established that NADAR family members can function as guanosine-ADP-ribosylhydrolases on RNA substrates.

### Phosphate RNA ADP-ribosylation is reversed by macrodomain- and ARH-like hydrolases

**Figure 2.**
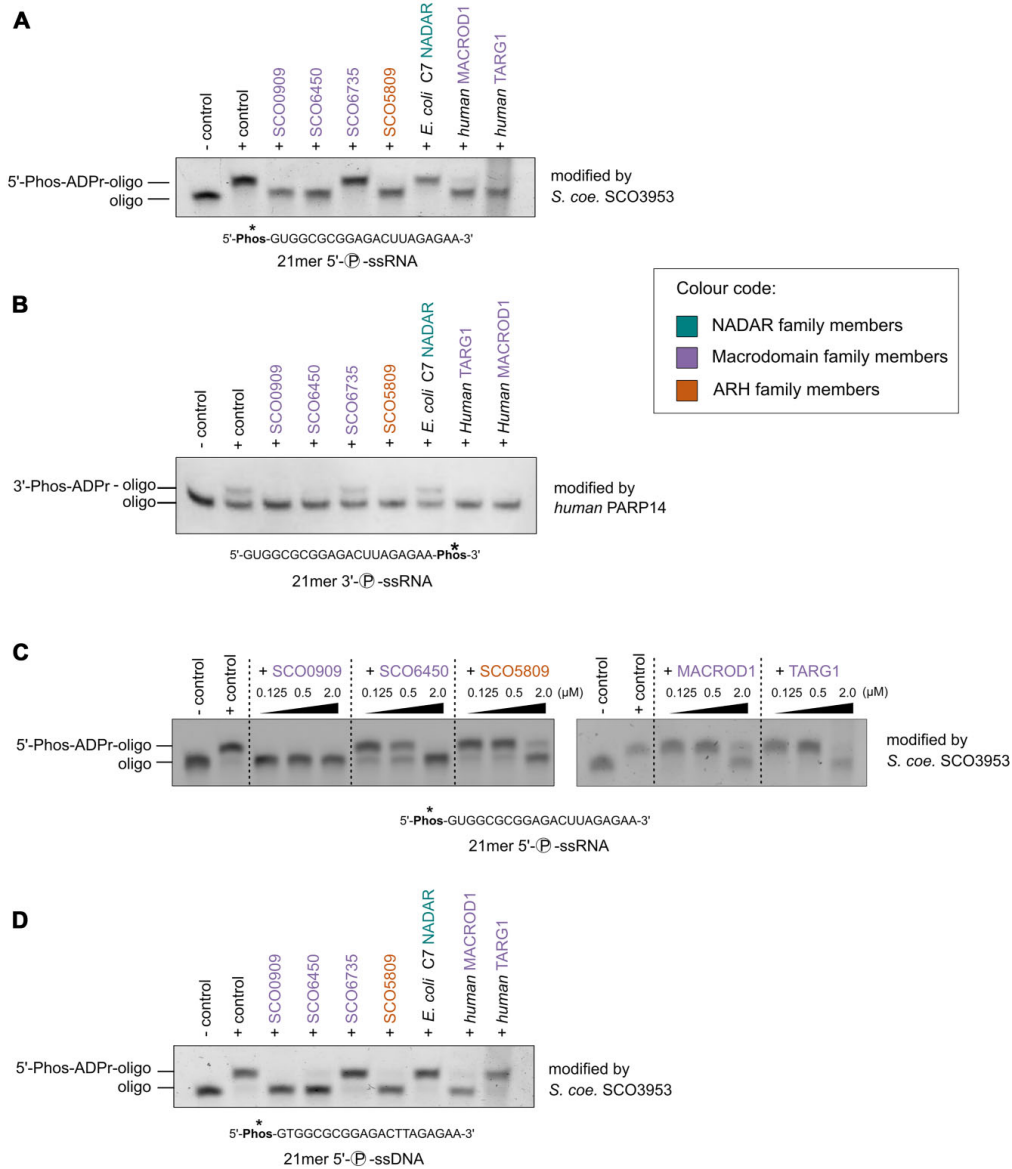
3′- and 5′-phosphate RNA ADP-ribosylation hydrolysis by macrodomain and ARH-like ADP-ribosylhydrolases. *In vitro* de-ADP-ribosylation assays showing hydrolytic activity of bacterial and human macrodomain, NADAR, and ARH-like enzymes on (**A**) 5′-phosphate single-stranded RNA (ssRNA) modified by *S. coelicolor* SCO3953 and (**B**) 3′-phosphate ssRNA modified by human PARP14. (**C**) Comparison of hydrolytic activities of macrodomain enzymes on *S. coelicolor* SCO3953 ADP-ribosylated 5′-phosphate ssRNA. Titration of ADP-ribosylhydrolases shows the higher de-ADP-ribosylation activity of *S. coelicolor* PARG SCO0909 compared with SCO6450 (MacroD-type macrodomain), SCO5809 (ARH-like) (left), and the human macrodomain hydrolases MacroD1 and TARG1 (right). (**D**) *In vitro* de-ADP-ribosylation assay showing hydrolytic activity of bacterial and human macrodomain, NADAR, and ARH-like enzymes on 5′-phosphate ssDNA modified by *S. coelicolor* SCO3953. The RNA/DNA oligonucleotide substrates are shown below each gel image with the site targeted for ADP-ribosylation/de-ADP-ribosylations highlighted with an asterisk. The colour code for NADAR, macrodomain, and ARH family members is shown in the inset.

To further understand the specificity of NADAR enzymes, we also analysed their ability to hydrolyse ADP-ribosylation modifications on the 5′- and 3′-phosphate groups on RNA ends. While some hydrolases have been shown to act on such substrates [38, 42], NADAR family members have not yet been analysed for this activity. Therefore, we enzymatically generated ADPr-phosphate-RNA using the Tpt1/KptA-like protein from *S. coelicolor* SCO3953 [38] for the 5′-end (Supplementary Fig. S2) and using human PARP14 for the 3′-end [38]. Analysis by gel-shift assays of *E. coli* C7 NADAR along with a panel of other bacterial and human hydrolases showed that NADARs were unable to process and de-modify RNA phosphate-end ADP-ribosylation. Yet, several hydrolases from macrodomain- and ARH-like families (*S. coelicolor* SCO0909, SCO6450, and SCO5809 as well as human MACROD1 and TARG1) reversed the ADP-ribosylation modifications from RNA on both phosphate ends (Fig. 2A and B) with various hydrolytic efficiencies (Fig. 2C). We also tested the capacity of each ADP-ribosylhydrolase to act on 5′-phosphate ADP-ribosylated DNA generated by either *S. coelicolor* SCO3953 (Fig. 2D) or by human TRPT1 [38] (Supplementary Fig. S3). As with 5′-phosphate-modified RNA, NADARs were unable to remove ADP-ribose from 5′-phosphate-modified DNA (Fig. 2D and Supplementary Fig. S3). Of note, the macrodomain family TARG1-like *S. coelicolor* SCO6735 that was also shown to be able to hydrolyse thymidine-linked ADP-ribosylation [48] which was proposed as a defence reaction against DarT-like toxins, was inactive on 5′-phospho-ADP-ribosylation on both, RNA and DNA. This is in contrast to human TARG1 which showed activity on these substrates, reversing 5′-phospho-ADP-ribosylation of RNA with higher efficiency compared with DNA (Fig. 2A and D). Yet, the highest hydrolytic activity on 5′-phospho ADPr-RNA was observed with bacterial PARG enzyme SCO0909 from *S. coelicolor* (Fig. 2C). Thus, NADAR enzymes appear to specifically remove guanosine base ADP-ribosylation, whereas ARH and macrodomain family enzymes are capable of hydrolysing phosphate-end modifications on RNA.

### PARGs reverse RhsP2-catalysed 2′-OH RNA ADP-ribosylation

**Figure 3.**
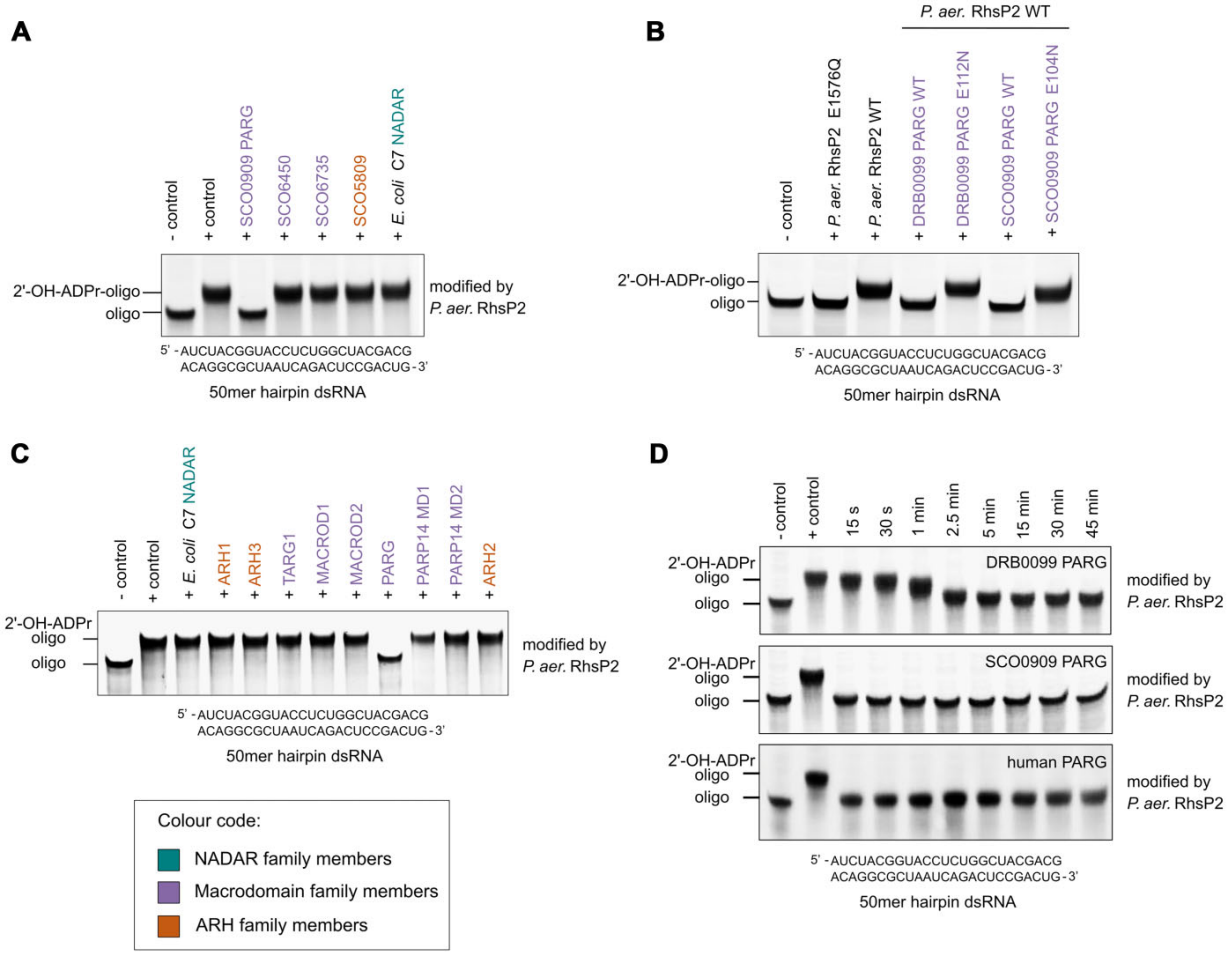
Reversal of RhsP2-catalysed 2′-OH RNA ADP-ribosylation by PARG enzymes. (**A**) *S. coelicolor* PARG SCO0909 reverses 2′-OH RNA ADP-ribosylation compared with *S. coelicolor* macrodomain and ARH-like enzymes and *E. coli* C7 NADAR. *In vitro* de-ADP-ribosylation assays were performed after modification of the dsRNA substrate by *P. aeruginosa* RhsP2, with the oligonucleotide sequence shown below the gel image. (**B**) Wild-type *S. coelicolor* PARG SCO0909 and *D. radiodurans* PARG DRB0099 reverse RhsP2-catalysed RNA ADP-ribosylation compared to catalytic mutants. dsRNA substrate is the same as shown in panel (A) below the gel image. (**C**) Human PARG reverses RhsP2-catalysed 2′-OH RNA ADP-ribosylation compared with human macrodomain- and ARH-like enzymes. dsRNA substrate is the same as shown in panel (A) below the gel. The colour code for NADAR, macrodomain, and ARH family members is shown in the inset bottom left. (**D**) Time course of 2′-OH RNA ADP-ribosylation hydrolysis by human and bacterial PARGs.

Another example of RNA ADP-ribosylation for which hydrolases were unknown has ADP-ribose linked to RNA at the 2′-OH of the ribose sugar moiety (Fig. 5). This RNA modification is catalysed by RhsP2, a toxin secreted by *P. aeruginosa* that kills competitor bacteria by interfering with essential host translation processes. In previous work, we showed that RhsP2 efficiently modifies dsRNA species and thus we used an RNA hairpin as a model substrate [40] for ADP-ribosylation using purified recombinant RhsP2. We then tested this RhsP2-modified RNA against our panel of bacterial ADP-ribosylhydrolases. Intriguingly, we found that only the PARG-like (bacterial-type PARG) enzyme *S. coelicolor* SCO0909 was able to reverse 2′-OH RNA ADP-ribosylation (Fig. 3A). To determine whether these findings could be generalized, we also examined a bacterial-type PARG from *Deinococcus radiodurans* (DRB0099), finding that it could also hydrolyse 2′-OH ADP-ribosylated RNA. Importantly, mutations in the PARG macrodomains targeting the main catalytic glutamate [50] abolished their hydrolytic activities (Fig. 3B). Of note, the distantly related TARG1-/ALC1-type macrodomain hydrolase [14] (SCO6735) and the MacroD-type macrodomain hydrolase (SCO6450) were unable to act on 2′-OH RNA ADP-ribosylation. Similarly, NADAR and ARH domain enzymes also exhibited no activity (Fig. 3A). Finally, we also tested a panel of human ADP-ribosylhydrolases against the RhsP2-modified RNA and found that only human PARG was capable of reversing 2′-OH RNA ADP-ribosylation (Fig. 3C). Time-course experiments (Fig. 3D) and enzyme titration experiments (Supplementary Fig. S4) found that SCO0909 was as efficient as human PARG at catalysing this hydrolysis and with higher activity compared with DRB0099. The observation is in line with previous studies showing that human PARG is also more efficient in poly-ADP-ribose (PAR) cleavage compared with *D. radiodurans* PARG, potentially indicating that human PARG is generally more active on ADP-ribose-related substrates than *D. radiodurans* PARG [51]. Thus, our data identify the first enzymes that are hydrolytically active on ribose hydroxyl group-linked RNA ADP-ribosylation and show that only the PARG-type of macrodomains [11] can catalyse this reaction.

### PARGs as immunity proteins protecting from RhsP2-mediated toxicity

**Figure 4.**
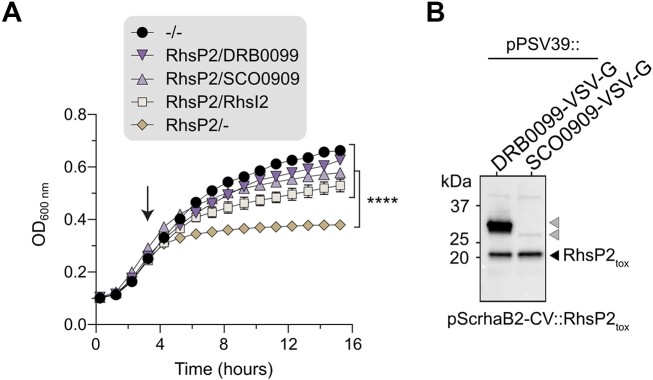
Bacterial PARGs as immunity proteins protect against RhsP2-mediated toxicity. (**A**) Liquid-culture growth curves of *E. coli* expressing RhsP2 toxin, and the indicated immunity protein (RhsI2 or *D. radiodurans* DRB0099 PARG or *S. coelicolor* SCO0909), or empty vector (-). Arrow indicates the timepoint when protein expression was induced. Data represent mean ± SD from three independently performed experiments (*N* = 3). ^****^P < 0.0001 determined by one-way ANOVA calculated with OD_600nm_ values at 15-h growth timepoint. (**B**) Western blot analysis showing protein levels after co-expression of *D. radiodurans* DRB0099 PARG or *S. coelicolor* SCO0909 with RhsP2_tox_ C-terminus in *E. coli* cells. Grey arrows indicate the bacterial PARG hydrolases.

PARGs and in particular bacterial PARG-type enzymes are found distributed in many bacterial species and evolutionary relationships indicate frequent horizontal gene transfer events. PARG enzymes are often not conserved in related bacterial species or even subspecies suggesting adaptation to the very specific environments [52]. Some bacteria code for multiple PARG enzymes, sometimes for both bacterial and canonical PARG types (Supplementary Fig. S5). The physiological roles of these enzymes in bacteria are unknown. However, given our findings that PARGs can efficiently reverse RhsP2-catalysed RNA ADP-ribosylation *in vitro*, we hypothesized that some bacterial PARGs might defend against toxic ARTs employed during microbial competition. Of note, a similar role was previously proposed for the SCO6735 macrodomain [48] which acts on thymidine ADP-ribosylation catalysed by DarT-like toxins. Thus, we next tested whether *S. coelicolor* SCO0909 or *D. radiodurans* DRB0099 PARGs could protect from RhsP2-induced toxicity *in vivo*. *P. aeruginosa* is protected from RhsP2 toxicity by RhsI2, a cognate immunity protein encoded downstream of *rhsP2* that neutralizes RhsP2 activity through the formation of a complex that occludes the toxin’s active site [40]. Remarkably, we found that co-expression of either *S. coelicolor* SCO0909 or *D. radiodurans* DRB0099 with RhsP2 in *E. coli* cells provided comparable protection against cellular intoxication to that of RhsI2 (Fig. 4A). Moreover, western blotting to measure expression levels of these PARGs found that *S. coelicolor* SCO0909 is expressed to considerably lower levels than that of RhsP2, suggesting that fewer relative copies of the hydrolase can nevertheless provide robust protection against RhsP2 (Fig. 4B). These data highlight the efficiency of SCO0909 at reversing the toxic products induced by RhsP2 *in vivo*. Altogether, we find that bacterial PARGs can act as defence enzymes against attacks of RhsP2-like toxins. These data suggest that many of the functionally enigmatic PARG enzymes found across diverse bacterial species may play critical roles in microbial immunity, particularly in defending against ADP-ribosylation-based attacks during microbial competition.

## Discussion

Nucleic acid ADP-ribosylation and its associated enzymes involved in catalysis and hydrolysis are widespread among all kingdoms of life [18, 29, 35, 53, 54], yet their roles in mammalian and bacterial physiology including inter-/intraspecies conflicts are only beginning to be unravelled. Recently, steadily more examples of enzymatic systems for RNA ADP-ribosylation have been identified, showing that all major types of RNA molecule species, including mRNA [55, 56], rRNA [57], and tRNA [40], can be ADP-ribosylated. ADP-ribosylation modifications of RNA can occur at various sites, including phosphates at the RNA ends [38], the N^2^ of the exocyclic amines of guanosine bases [39, 56], and the ribose 2′-OH of the RNA backbone [40]. Furthermore, the attachment of ADP-ribose at adenines at position N^6^ in GA dinucleotides in mRNA was very recently suggested to be catalysed by the ART toxin CmdT which is part of the toxin–antitoxin–chaperone system CmdTAC. Modifications by CmdT can block translation and represent a novel mechanism of anti-phage defence [55, 56]. Noteworthy, in some instances, as for the *Photorhabdus laumondii* toxin Tre23, that after its delivery by type VI secretion systems into the target cell ADP-ribosylates ribosomal 23S RNA, the exact molecular linkage of the ADP-ribose to the RNA has not been identified yet [57], potentially hinting at the discovery of an undescribed type of RNA-ADP-ribosyl modification reaction.

While the reversal of thymidine and guanosine ADP-ribosylation modifications on DNA has recently been described [17, 29, 34, 36], such reversibility has not yet been investigated for RNA ADP-ribosylation modifications on RNA bases and hydroxyl groups. Here, we demonstrate that both can be reversed by hydrolyses belonging to the NADAR enzyme and PARG-type macrodomain family, respectively. The ability of NADARs to reverse ScARP-catalysed guanosine ADP-ribosylation on RNA substrates expands the known activity profile of NADAR enzymes and suggests they may be used by both bacteria and eukaryotes to protect against toxic attacks that could interfere with host translation processes. Furthermore, the observation that RNA ADP-ribosylation is reversible may indicate that such RNA modifications may have endogenous functions akin to those observed for the reversible DNA-ADPr [27], thereby regulating important undiscovered cellular processes.

**Figure 5.**
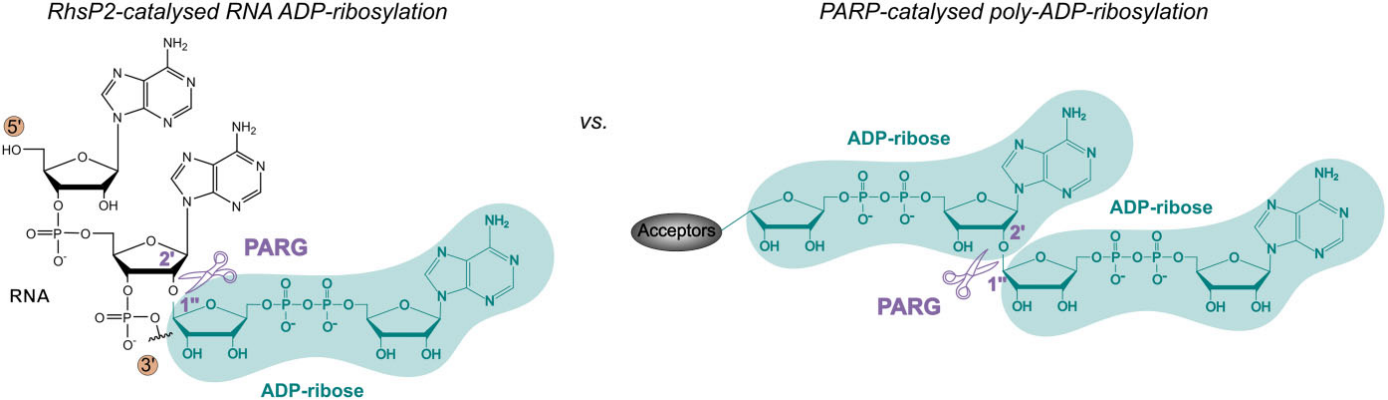
Comparison of chemical ADP-ribosyl linkages catalysed by RhsP2 on RNA and PARPs in a process called ‘poly-ADP-ribosylation’. RhsP2 and PARPs establish the same ADP-ribose linkage between the 2′-OH of the ribose sugar and the C1’’ of the ADP-ribose which can be hydrolysed by PARG.

We also demonstrate that the bacterial PARG-type enzymes from *D. radiodurans* and *S. coelicolor* [50, 58] efficiently remove ADP-ribose adducts from ribose 2′-OH in RNA. We discovered this enzyme activity by using as a model system the effector toxin RhsP2 toxin secreted by *P. aeruginosa* that ADP-ribosylates 2′-OH in dsRNA. The pathogen has its own immunity protein, RhsI2, which protects the organism from RhsP2 toxic effects by complex formation with RhsP2 [40]. Yet, RhsI2 is confined to the host and thus, PARGs that enzymatically counteract RhsP2 toxic activity may function as protectors of competitor bacteria against RhsP2-like toxins. Indeed, we observed efficient protection against RhsP2-mediated toxicity by SCO0909 and DRB0099 *in vivo* which is in line with the observed *in vitro* activities of these enzymes. Noteworthy, the molecular linkage established by RhsP2 between the anomeric carbon (C1’’) of the distal ribose of the ADP-ribose and the 2′-OH in the RNA sugar-phosphate backbone is identical to the linkage between repeating ADP-ribose units within PAR chains (Fig. 5). Human PARGs are characterized for their processing activity of PAR to ADP-ribose monomers by cleaving this linkage [50, 59], rationalizing the ability of the bacterial PARG homologues to hydrolyse the RhsP2-catalysed RNA-ADPr adducts.

**Figure 6.**
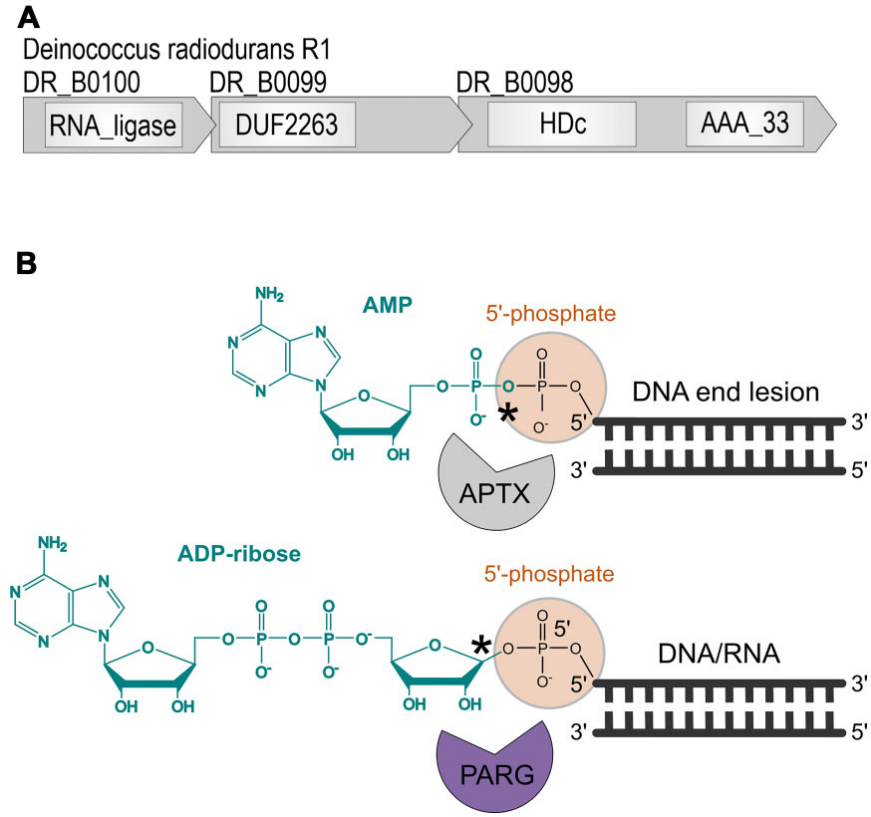
PARG as a repair protein for ADP-ribose blocked DNA/RNA ends. (**A**) *D. radiodurans* PARG (DRB0099) is part of a putative three-gene operon involved in DNA repair. (**B**) Comparison between APTX and PARG with their hydrolytic activity to resolve AMP- and ADP-ribose blocked DNA/RNA 5′-phosphate ends, respectively. The hydrolysed linkage by the respective enzyme is highlighted with an asterisk.

In our study, we explore so far undescribed enzymatic reactions on RNA ADP-ribosylation, and it is conceivable that RNA ADP-ribosylation has important physiological functions in *S. coelicolor* and *D. radiodurans*. Interestingly, the gene coding for *D. radiodurans* PARG (DRB0099) is part of a putative three-gene operon (Fig. 6A) [60] whose transcription is induced after DNA damage [61] and which has a proposed role in RNA and/or DNA repair [60]. The first gene in the operon, DRB0100, codes for an RNA/DNA ligase that is followed by PARG and the third gene DRB0098. The latter possesses two domains, an HD-phosphohydrolase family phosphatase domain and an active polynucleotide kinase domain phosphorylating 5′-OH termini [60]. As such, it has predicted biochemical activity analogous to that of the human PNKP protein that plays an important role in DNA break repair via the processing of phosphate groups at DNA ends [62, 63]. As we demonstrated, bacterial-type PARGs show high hydrolytic activity on ADP-ribosylated 5′-phosphate ends on RNA/DNA which was catalysed using human TRPT1 and SCO3953, the bacterial equivalent Tpt1/KptA-like ART [38], as model enzymes. It is therefore conceivable that bacterial encoded PARG has its role as a repair enzyme of ADP-ribosylated phosphate ends produced by KptA/TpT1-like toxins ADP-ribosylating such ends as their mechanism of action. If so, this ADPr-phosphate processing activity on DNA/RNA by bacterial PARGs would resemble the action of DNA end-processing repair factor aprataxin (APTX) that removes adenylate adducts from 5′-phosphate groups in DNA and RNA [64, 65]. Together with their hydrolytic activity on 2′-OH–C1’’ linkages described above, PARGs thus accept an expanded substrate range, potentially making them more universally protective enzymes against toxic RNA ADP-ribosylation adducts (Fig. 6B).

Finally, another example of an ADP-ribosylhydrolase potentially employed as defence protein against toxic attacks is the *S. coelicolor* SCO6735. The TARG1 macrodomain-like enzyme, which is also induced by DNA damage [66], is hydrolytically active on ADP-ribosylation marks on RNA phosphate ends (this study) and DNA thymidine bases [48]. Thus, our findings suggest that one of the ways in which SCO6735 protects DNA/RNA from damage is by removing phosphate or thymine-linked ADP-ribosylation caused by Tpt1/KptA- and DarT-like exotoxins [48], respectively.

Altogether, our findings expand our insights on the seemingly widespread occurrence and reversal of RNA ADP-ribosylation. Yet apart from recent studies on NADARs, the physiological functions of bacterial PARGs, macrodomain enzymes (SCO6450 and SCO6735), and ARH enzymes (SCO5809) showing hydrolytic activities on RNA ADP-ribosylated substrates are unexplored and our presented findings will provide starting points for future studies. The discovery of potential novel defence modules sheds light on the variety of inter-/intraspecies competition warfare strategies, but it may also facilitate discovery of novel pathways controlled by endogenous reversible ADP-ribosylation of RNA as shown for DNA ADP-ribosylation [27]. Understanding the activities, interactions, and functions of the enzymatic key players may also identify new targets for antimicrobial approaches, as suggested for targeting DarG antitoxins [67].

## Supplementary Material

gkaf069_Supplemental_File

## Data Availability

The data underlying this article are available in the article and in its online supplementary material.
