# Supplementary material for: Discovery of reversing enzymes for RNA ADP-ribosylation reveals a possible defence module against toxic attack
Source: Nucleic Acids Res. 2025 Feb 18;53(4):gkaf069. doi: 10.1093/nar/gkaf069 (PMC11833690; doi:10.1093/nar/gkaf069)
Supplement: gkaf069_Supplemental_File [file gkaf069_supplemental_file.pdf]

Discovery of reversing enzymes for RNA ADP-ribosylation reveals a possible defence module against toxic attack

Yang Lu[1‡], Marion Schuller[1‡,*], Nathan P. Bullen[2‡], Petra Mikolcevic[3], Iva Zonjic[3], Roberto Raggiaschi[1], Andreja Mikoc[3], John C. Whitney[2,*], Ivan Ahel[1,*]

[1]*Sir William Dunn School of Pathology, University of Oxford, Oxford, United Kingdom*
[2]*Department of Biochemistry & Biomedical Sciences, McMaster University, Hamilton, Ontario, Canada*
[3]*Division of Molecular Biology, Ruđer Bošković Institute, Zagreb, Croatia*

‡ These authors contributed equally.

* Corresponding authors:
marion.schuller@path.ox.ac.uk, jwhitney@mcmaster.ca, and ivan.ahel@path.ox.ac.uk

Supplementary Information

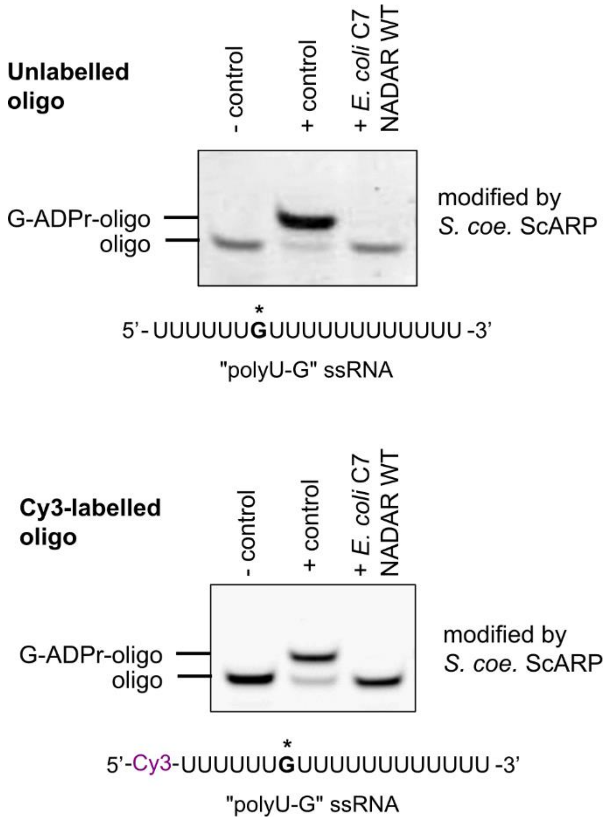

Supplementary Figure S1 [related to Figure 1]. ADP-ribose modification enhances staining of polyU-G oligonucleotides by SYBR- dye. Comparison of visualisation of Cy3-labelled oligo (bottom) (which does not rely on SYBR dye staining) with unlabelled oligo (top) (visualised with SYBR dye). In case of the Cy3-labelled oligo, the unmodified and ADP-ribosylated oligo visualise similarly. This is in contrast with the unlabelled oligo. The presence of the ADP-ribose modification enhances the staining of the oligo and its visualisation.

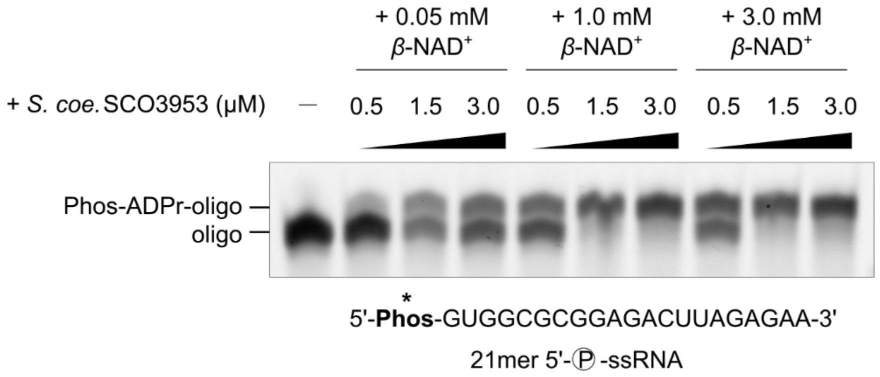

Supplementary Figure S2 [related to Figure 2]. Establishing enzyme and substrate for 5'-phospho RNA ADP-ribosylation. Titration of *S. coelicolor* SCO3953 against 5'-phosphorylated RNA substrate. An β-NAD$^+$ of 1 mM and enzyme concentration of 1.5 µM resulted in complete ADP-ribosylation of the RNA substrate. These conditions were used for subsequent hydrolase activity testing.

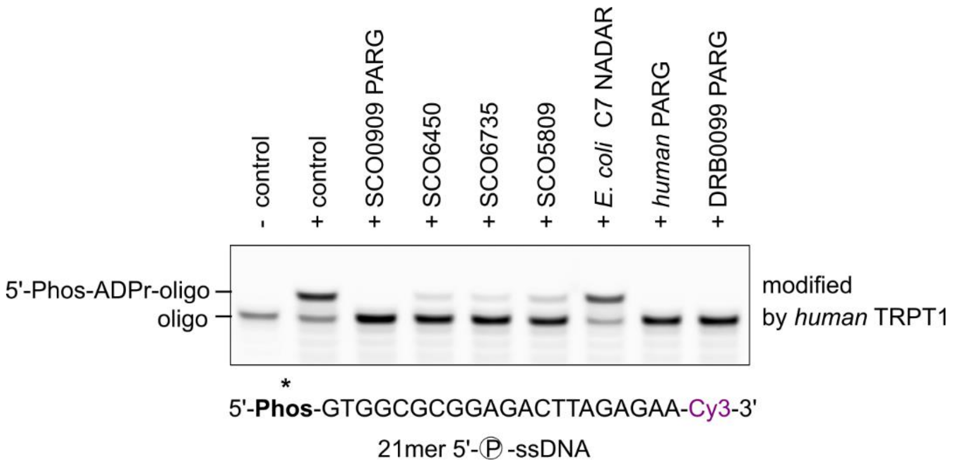

Supplementary Figure S3 [related to Figure 2]. 5'-phosphate DNA ADP-ribosylation hydrolysis by macrodomain enzymes. *In vitro* de-ADP-ribosylation assay using a 5'-phosphate DNA oligonucleotide substrate ADP-ribosylated by human TRPT1 showing hydrolytic activity of bacterial and human macrodomain enzymes compared to NADAR family members. Figure relates to Figure 2a showing activity of the enzymes on the corresponding RNA substrate.

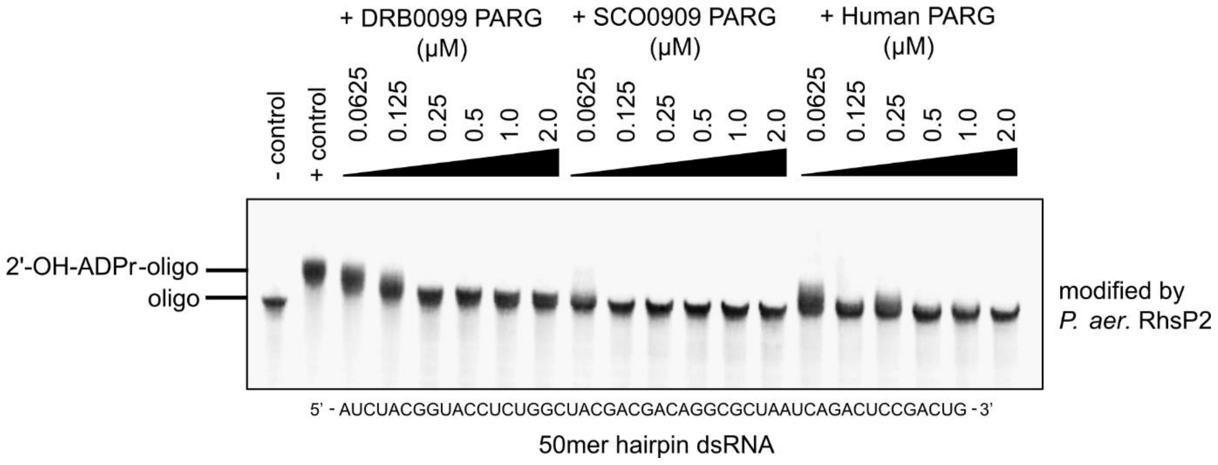

Supplementary Figure S4 [related to Figure 3]. Comparison of hydrolysis efficiencies of bacterial and human PARG enzymes of 2'hydroxyl ADP-ribose RNA. Concentration titrations reveal that *D. radiodurans* DRB0099 PARG is less efficient than *S. coelicolor* SCO0909 PARG and human PARG.

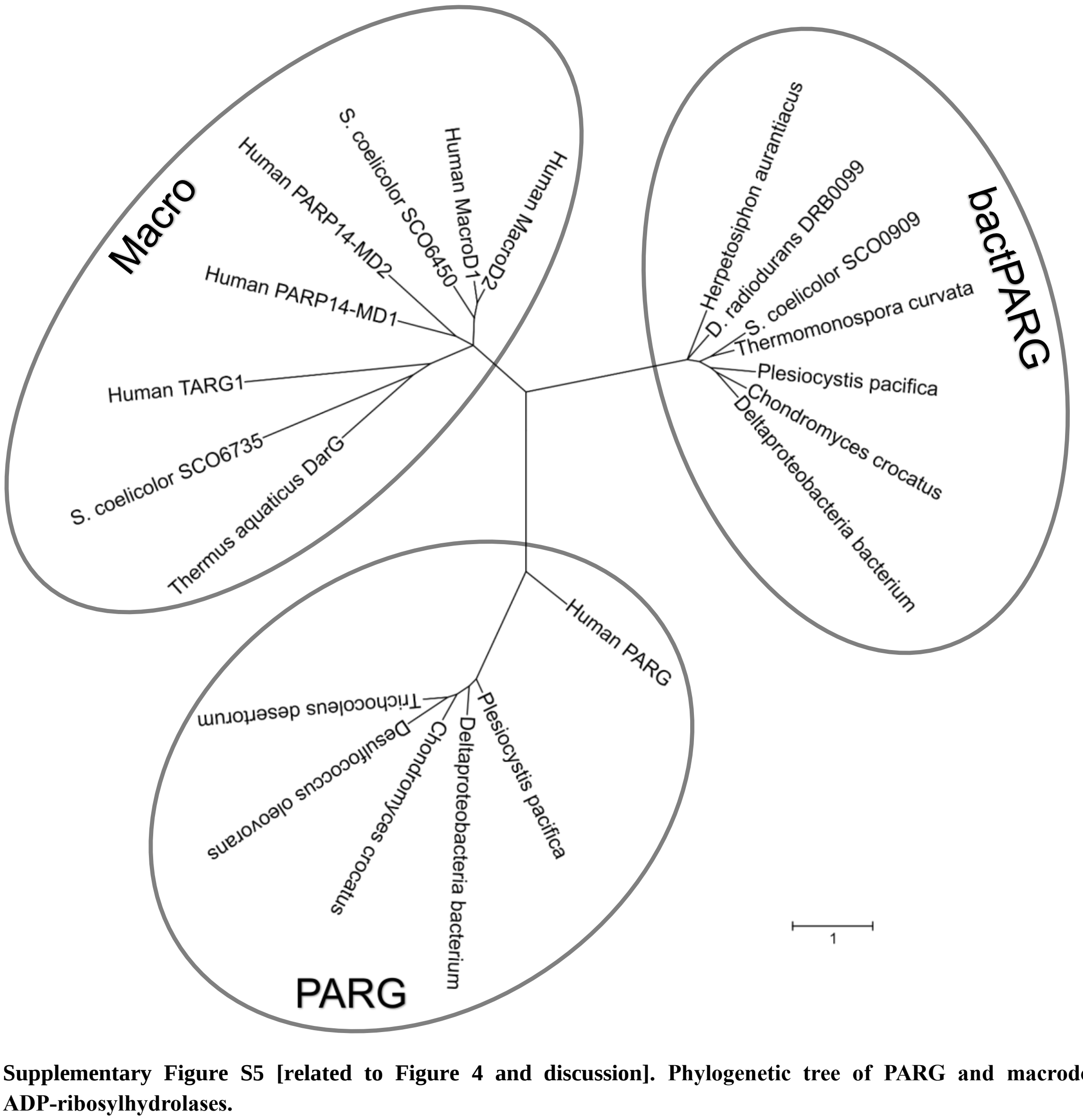

Supplementary Figure S5 [related to Figure 4 and discussion]. Phylogenetic tree of PARG and macrodomain ADP-ribosylhydrolases.

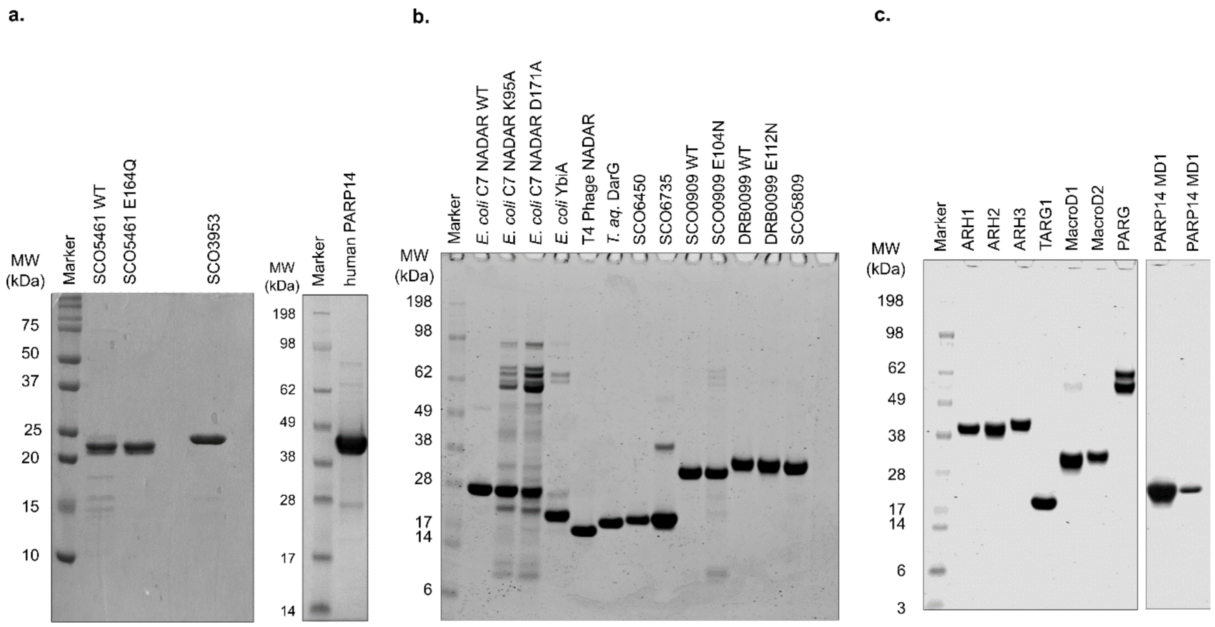

Supplementary Figure S6. Uncropped SDS-PAGE gel images of (a) bacterial and human transferases, (b) bacterial hydrolases and c) human hydrolases. 2 µg of protein was analysed alongside a protein marker.

Figure 1 a

Experiment 1

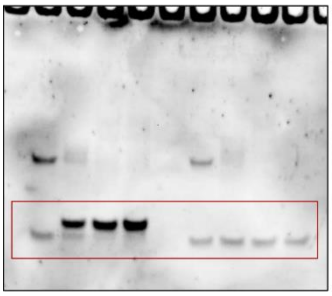

Experiment 2

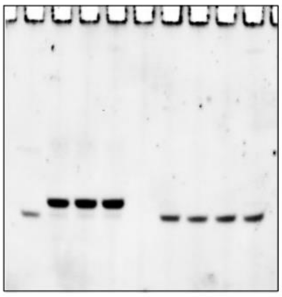

Figure 1 b

Experiment 1

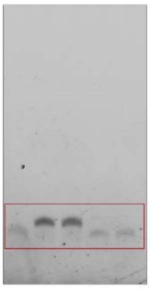

Experiment 2

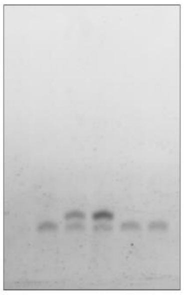

Figure 1 c

Experiment 1

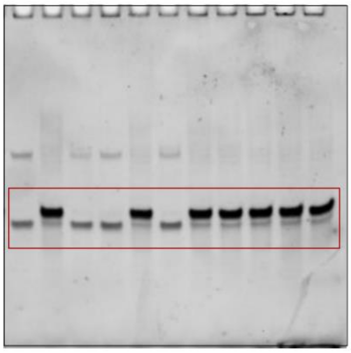

Experiment 2

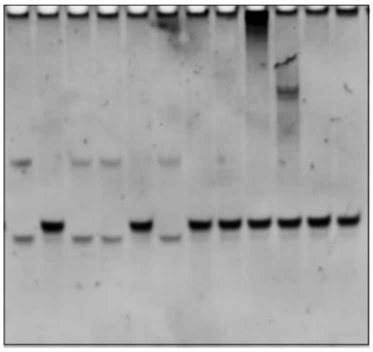

Figure 1 d

Experiment 1

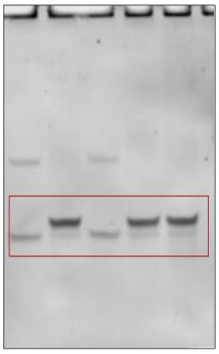

Experiment 2

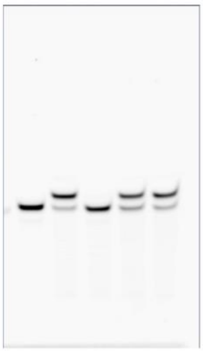

Figure 1 e

Experiment 1

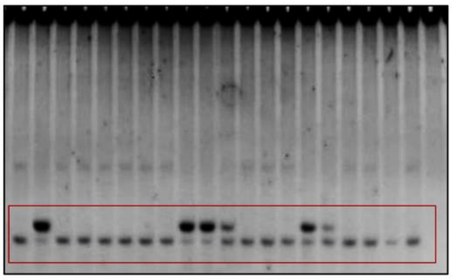

Experiment 2

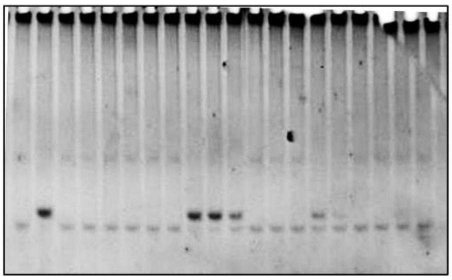

Figure 1 f

Experiment 1

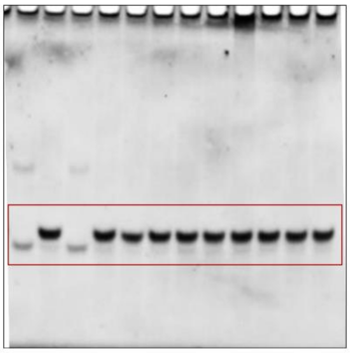

Experiment 2

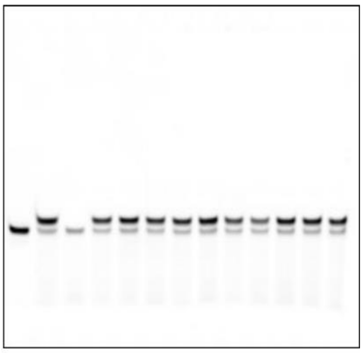

Supplementary Figure S7-1. Uncropped gels from gel-shift assays of Figure 1. Data presented in the manuscript are highlighed with a red box.

Figure 2 a

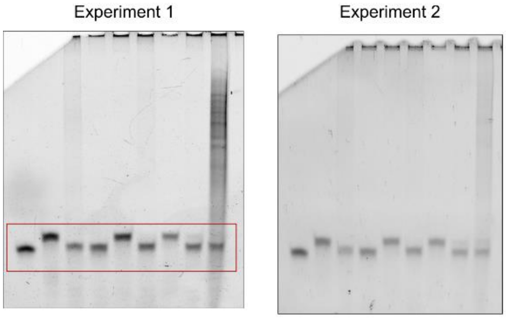

Figure 2 b

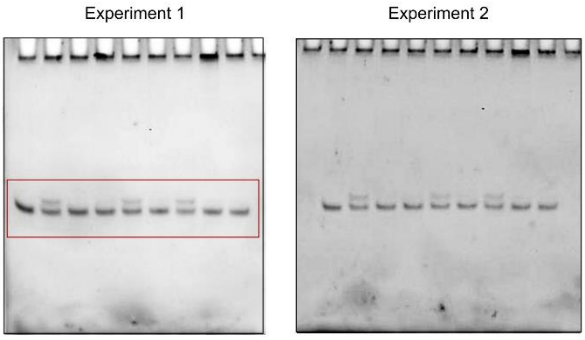

Figure 2 c

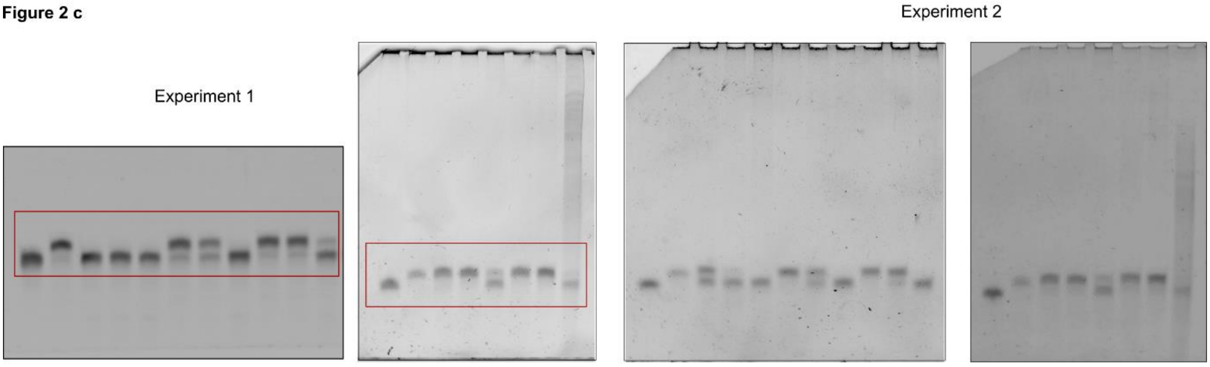

Figure 2 d

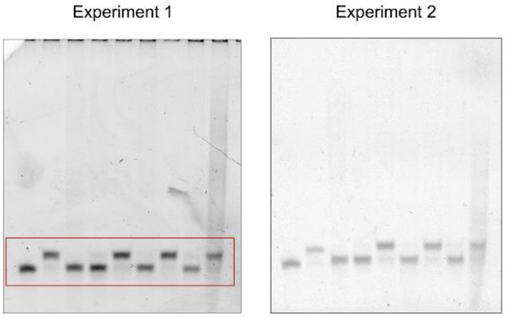

Supplementary Figure S7-2. Uncropped gels from gel-shift assays of Figure 2. Data presented in the manuscript are highlighed with a red box.

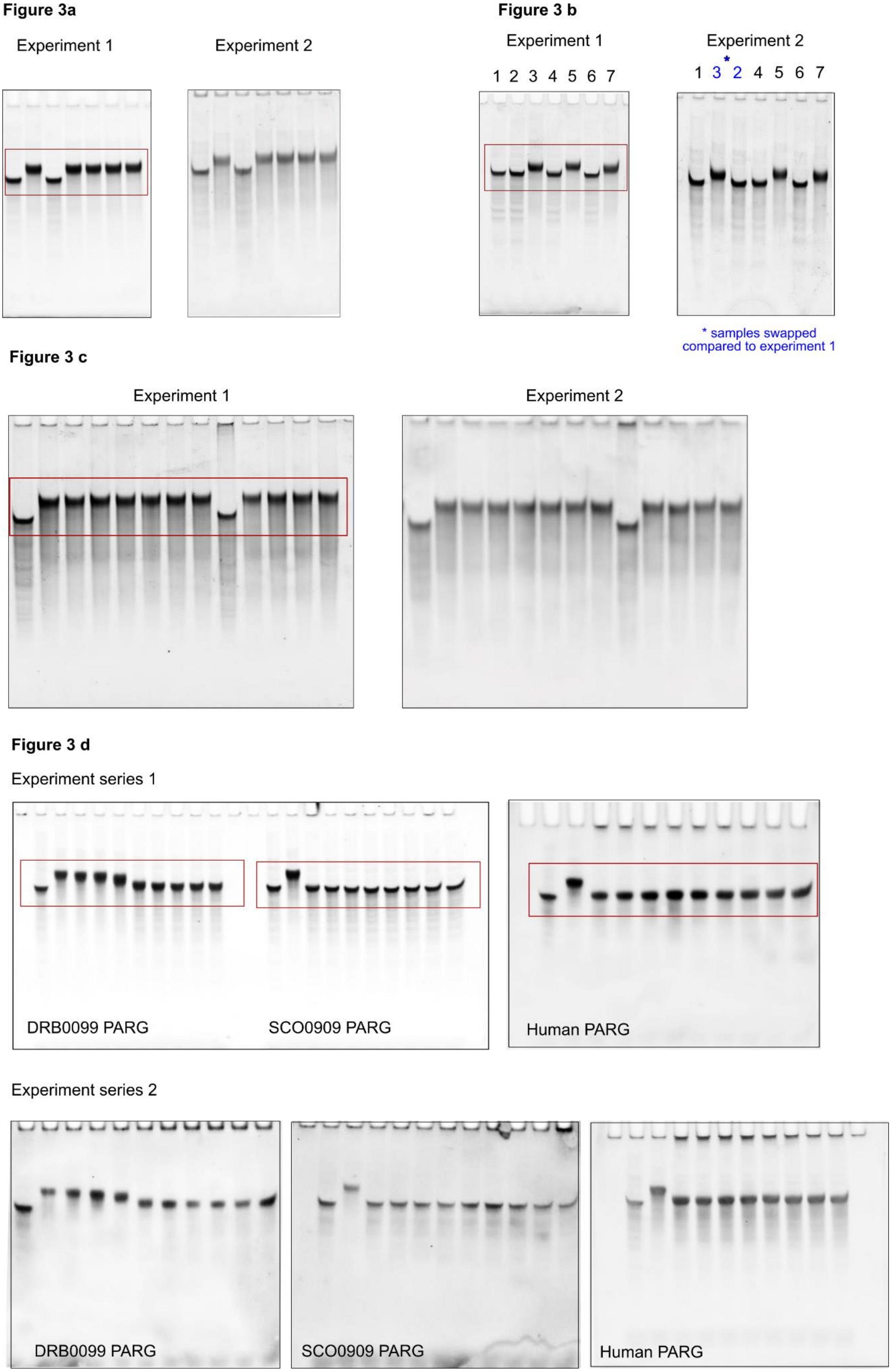

Supplementary Figure S7-3. Uncropped gels from gel-shift assays of Figure 3. Data presented in the manuscript are highlighed with a red box.

SI Figure S3

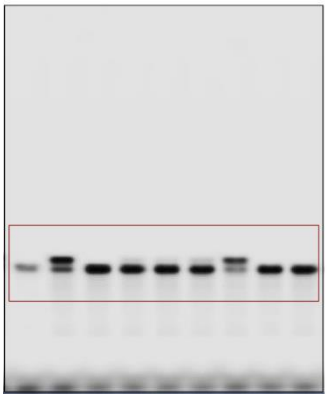

SI Figure S4

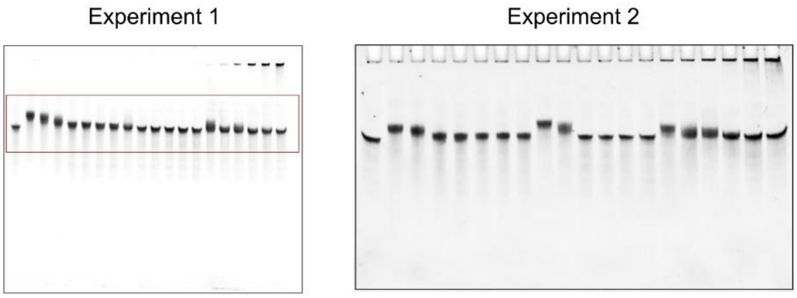

Supplementary Figure S7-4. Uncropped gels from gel-shift assays from Supplementary Figures. Data presented in the manuscript are highlighed with a red box.

Supplementary Table 1. Primers and substrates used in this study. Relates to Materials and Methods.

Oligonucleotide	Description	Sequence (5’→3’)
SCO0909_Forward_RBS_Xbal	SCO0909 primer for cloning into pPSV39	TGTTAAGCTATCTAGAACGGGAGAAAGATGAGCGCGCGCCTGCGGGG
SCO0909_Reverse_HindIII	SCO0909 primer for cloning into pPSV39	TGTTAAGCTAAAGCTTCTACGGCTGGACCTGACCTGG
DRB0099_Forward_RBS_Xbal	DRB0099 primer for cloning into pPSV39	TGTTAAGCTATCTAGAACGGGAGAAAGATGAACCGCAAAAACCGTACCGAACAG
DRB0099_Reverse_HindIII	DRB0099 primer for cloning into pPSV39	TGTTAAGCTAAAGCTTTCAGGAGGTAGATGAGGGCAGACATA
DRB0099_Forward_RBS_EcoRI	DRB0099 primer for cloning into pPSV39-CV	TGTTAAGCTAGAATTCACGGGAGAAAGATGAACCGCAAAAACCGTACCGAACAG
DRB0099_Reverse_NoStop_Xbal	DRB0099 primer for cloning into pPSV39-CV	TGTTAAGCTATCTAGAGGAGGTAGATGAGGGCAGACATAAGGA
SCO0909_Forward_RBS_EcoRI	SCO0909 primer for cloning into pPSV39-CV	TGTTAAGCTAGAATTCACGGGAGAAAGATGAGCGCGCGCCTGCGGGG
SCO0909_Reverse_NoStop_Xbal	SCO0909 primer for cloning into pPSV39-CV	TGTTAAGCTATCTAGACGGCTGGACCTGACCTGGGCG
50mer RNA oligo	RhsP2 substrate	AUCUACGGUACCUCUGGCUACGACGACAGGCGCUAAUCAGA CUCCGACUG
PolyU-G	ScARP substrate	UUUUUUGUUUUUUUUUUU
PolyU-A	ScARP substrate	UUUUUUAUUUUUUUUUUU
5’-Phos-ssDNA	SCO3953 DNA substrate	[Phos]-GTGGCGCGGAGACTTAGAGAA
5’-Phos-ssDNA-Cy3	TRPT1 DNA substrate	[Phos]-GTGGCGCGGAGACTTAGAGAA-Cy3
5’-Phos-ssRNA	SCO3953 RNA substrate	[Phos]-GUGGCGCGGAGACUUAGAGAA
3’-Phos-ssRNA	PARP14 RNA substrate	GUGGCGCGGAGACUUAGAGAA-[Phos]

Supplementary Table 2. Recombinant DNA plasmids for bacterial toxicity assays. Relates to Materials and Methods.

Plasmid	Relevant Features	Reference
pSCrhaB2-CV	Expression vector with PrhaB, C-terminal VSV-G tag, TmpR	Cardona and Valvano, 2005 (link)
pSCrhaB2CV::PA14_43100_1445CT_Y1524A-VSV-G	Expression vector for C-terminal VSV-G tagged rhsP2tox Y1524A	Bullen et al., 2022 (link)
pPSV39-CV	Expression vector with lacI, lacUV5 promoter, C-terminal VSV-G tag, GmR	Silverman et al., 2013 (link)
pPSV39-CV::PA14_RS30650	Expression vector for rhsI2	Bullen et al., 2022
pPSV39-CV::SCO0909	Expression vector for SCO0909	This Study
pPSV39-CV::DRB0099	Expression vector for DRB0099	This Study
pPSV39-CV::SCO0909-VSV-G	Expression vector for SCO0909 harboring a C-terminal VSV-G tag	This Study
pPSV39-CV::DRB0099-VSV-G	Expression vector for DRB0099 harboring a C-terminal VSV-G tag	This Study
pPSV39-CV::RhsI2	Expression vector for RhsI2	Bullen et al., 2022 (link)
pET28_SC_*SCO5461*	pET28a carrying *S. coelicolor scarp (SCO5461),* aa 35-204; kan^{R}	Lalic et al., 2016 (link)
pET28_SC_*SCO5461*^{E164Q}	pET28a carrying *S. coelicolor scarp (SCO5461),* aa 35-204; kan^{R}	Lalic et al., 2016 (link)
pET28_SC_*SCO3953*	pET15bcarrying *S. coelicolor transferase (SCO3953),* full-length; amp^{R}	Munnur et al., 2019 (link)
pNIC28_HS_PARP14	pNIC28-Bsa4 carrying *human* PARP14 catalytic-WWE domain, aa 1459-1801; kan^{R}	Munnur et al., 2019 (link)
pET28_Ecoli_*nadar*	pET28a carrying *E. coli* C7 *nadar* full-length; kan^{R}	Schuller et al., 2023 (link)
pET28_Ecoli_*nadar*^{K95A}	pET28a carrying *E. coli* C7 *nadar*^{K95A} full-length; kan^{R}	Schuller et al., 2023 (link)
pET28_Ecoli_*nadar*^{D171N}	pET28a carrying *E. coli* C7 *nadar*^{D171N}full-length; kan^{R}	Schuller et al., 2023 (link)
pET_Ecoli_*ybia*	pET28a carrying *E. coli* K-12 *ybiA* full-length; kan^{R}	Cihlova et al., 2024 (link)
pET28_T4_*nadar*	pET28a carrying *E. coli* T4 phage (gp30.3) *nadar* full-length; kan^{R}	Cihlova et al., 2024 (link)

pET28_Taq_*darG*_macro	pET28a carrying *T. aquaticus darG* macrodomain (aa 1-155); kan^R	Jankevicius *et al.*, 2017 (link)
pET28_SC_*SCO6450*	pET15b carrying *S. coelicolor* hydrolase *(SCO6450)*, full-length; amp^R	Agnew et al., 2018 (link)
pET28_SC_*SCO6735*	pET15b carrying *S. coelicolor* hydrolase *(SCO6735)*, full-length; amp^R	Lalic et al., 2016 (link)
pET28_SC_*SCO5809*	pET28b carrying *S. coelicolor* hydrolase *(SCO5809)*, full-length; kan^R	This study
pET_SC_SCO0909	pET28b carrying *S. coelicolor PARG (SCO0909)*, wild-type, full-length; kan^R	This study
pET_SC_$SCO0909^{E112N}$	pET28b carrying *S. coelicolor PARG* ($SCO0909^{E112N}$), full-length; kan^R	This study
pET_DR_SCO0909	pET28b carrying *D. radiodurans* PARG DRB0099, wild-type, full-length; kan^R	This study
pET_DR_$SCO0909^{E104N}$	pET28b carrying *D. radiodurans* PARG DRB0099 ($SCO0909^{E104N}$), full-length; kan^R	This study
pDEST_HS_ARH1	pDEST17 carrying *H. sapiens* ARH1, full-length; amp^R	Fontana et al., 2017 (link)
pDEST_HS_ARH2	pDEST17 carrying *H. sapiens* ARH2, full-length; amp^R	Fontana et al., 2017 (link)
pDEST_HS_ARH3	pDEST17 carrying *H. sapiens* ARH3, full-length; amp^R	Rack et al., 2021 (link)
pDEST_HS_TARG1	pDEST17 carrying *H. sapiens* TARG1, full-length; amp^R	Sharifi et al., 2013 (link)
pETM_HS_MacroD1	pETM-CN carrying *H. sapiens* MacroD1, aa 91-235; kan^R	Jankevicius et al., 2013 (link)
pETM_HS_MacroD2	pET28a carrying *H. sapiens* MacroD2, aa aa 7-243; kan^R	Jankevicius et al., 2013 (link)
pET_HS_PARG	pET28a carrying *H. sapiens* PARG, 448-976; kan^R	Dunstan et al., 2012 (link)
pET_HS_PARP14 MD1	pET28a carrying *H. sapiens* PARP14 MD1, aa 798-979; kan^R	Dukic et al., 2023 (link)
pET_HS_PARP14 MD2	pET28a carrying *H. sapiens* PARP14 MD2, aa 994-1191; kan^R	Dukic et al., 2023 (link)

Supplementary Table 3. Bacterial strains used in this study. Relates to Materials and Methods.

Strain	Genotype	Description	Reference/Source
E. coli XL-1 Blue	*recA1 endA1 gyrA96 thi-1 hsdR17 supE44 relA1 lac* [F´ *proAB lacIq* ZΔ*M15* Tn*10* (Tet^R)]	Cloning strain	Novagen
Rosetta^{TM} BL21 (DE3)	*F-ompT hsdSB(rB- mB-) gal dcm (DE3) pRARE* (cam^R)	Expression strain	Novagen
